# Local host response following an intramammary challenge with *Staphylococcus fleurettii* and different strains of *Staphylococcus chromogenes* in dairy heifers

**DOI:** 10.1186/s13567-016-0338-9

**Published:** 2016-05-12

**Authors:** Kristine Piccart, Joren Verbeke, Anneleen De Visscher, Sofie Piepers, Freddy Haesebrouck, Sarne De Vliegher

**Affiliations:** M-team and Mastitis and Milk Quality Research Unit, Department of Reproduction, Obstetrics, and Herd Health, Faculty of Veterinary Medicine, Ghent University, Salisburylaan 133, 9820 Merelbeke, Belgium; Department of Pathology, Bacteriology and Avian Diseases, Faculty of Veterinary Medicine, Ghent University, Salisburylaan 133, 9820 Merelbeke, Belgium

## Abstract

Coagulase-negative staphylococci (CNS) are a common cause of subclinical mastitis in dairy cattle. The CNS inhabit various ecological habitats, ranging between the environment and the host. In order to obtain a better insight into the host response, an experimental infection was carried out in eight healthy heifers in mid-lactation with three different CNS strains: a *Staphylococcus fleurettii* strain originating from sawdust bedding, an intramammary *Staphylococcus chromogenes* strain originating from a persistent intramammary infection (*S. chromogenes* IM) and a *S. chromogenes* strain isolated from a heifer’s teat apex (*S. chromogenes* TA). Each heifer was inoculated in the mammary gland with 1.0 × 10^6^ colony forming units of each bacterial strain (one strain per udder quarter), whereas the remaining quarter was infused with phosphate-buffered saline. Overall, the CNS evoked a mild local host response. The somatic cell count increased in all *S. fleurettii*-inoculated quarters, although the strain was eliminated within 12 h. The two *S. chromogenes* strains were shed in larger numbers for a longer period. Bacterial and somatic cell counts, as well as neutrophil responses, were higher after inoculation with *S. chromogenes* IM than with *S. chromogenes* TA. In conclusion, these results suggest that *S. chromogenes* might be better adapted to the mammary gland than *S. fleurettii*. Furthermore, not all *S. chromogenes* strains induce the same local host response.

## Introduction

Coagulase-negative staphylococci (CNS) are the principal cause of subclinical mastitis in dairy cattle [1], especially in primiparous cows [2]. The impact of CNS on the udder health of dairy cattle has gained more attention in the past decade. The CNS were initially reported as one large, uniform group of bacteria [3, 4]. However, thanks to recent advances in molecular identification techniques, individual CNS species have become easier to identify and study [5, 6]. Over 20 different CNS species have been isolated from bovine milk [7]. Nonetheless, the bovine-associated CNS cover a wide range of ecological habitats, varying from essentially environmental species to host-adapted species [8]. Some CNS species rarely occur in bovine milk, but rather thrive in the environment of the cow and the barn (e.g. air, sawdust, bedding, and floors) [9]. These so-called environmental CNS species include, among others, *Staphylococcus equorum* and *Staphylococcus fleurettii* [8]. On the other end of the spectrum are the so-called host-adapted CNS species, specialized in survival in the udder and on the cow. *Staphylococcus chromogenes* is considered such a species [10], since it is the predominant CNS species found in milk [8, 11]. Furthermore, *S. chromogenes* is also present on the teat apex [12, 13], streak canal, and other extra-mammary body sites [14]. *Staphylococcus chromogenes* is one of the main CNS species involved in intramammary infections (IMI) [15, 16]. In general, *S. chromogenes* causes a minor to moderate increase in the milk somatic cell count (SCC) [17], although one study noted a rise in SCC comparable to that of *S. aureus* infections [18]. On the other hand, teat apex colonization with *S. chromogenes* has also been associated with a lower quarter milk SCC in early lactating heifers [12], whereas some strains can even inhibit the growth of other mastitis pathogens in vitro [19].

Epidemiological data suggest that not all CNS species exhibit the same degree of pathogenicity [18], but little is known about the different host responses caused by an IMI with representatives of the supposed environmental or host-adapted species (or strains). Therefore, the first objective of the research was to examine the host response and bacterial shedding following an experimental intramammary inoculation in heifers with one distinctive host-adapted CNS species and another typical environmental one (*S. chromogenes* versus *S. fleurettii*). The second objective was to evaluate whether the elicited host response and bacterial shedding differs between strains belonging to the same species, in this case *S. chromogenes*.

## Materials and methods

The study is in compliance with the European Directive 2010/63/EU, and was approved by the Ethics Committee of the Faculty of Veterinary Medicine, Ghent University (EC2012/73).

### Animals

The experiment was performed at the research dairy farm of Ghent University (Biocentrum Agri-Vet, Melle, Belgium). Eight clinically healthy Holstein–Friesian heifers in mid-lactation (78–278 days in milk) were selected. Heifers with a previous history of clinical mastitis or persistent high SCC (>150 000 cells/mL) on Dairy Herd Improvement records were not included. To increase the likelihood that all quarters were free from IMI, the animals received 15 days before the start of the experiment 3 daily intramuscular injections of 10 g penethamate hydroiodide (Mamyzin, Boehringer Ingelheim GmbH) combined with an intramammary treatment of 200 mg cephalexin and 100 000 I.U. kanamycin (Ubrolexin, Boehringer Ingelheim GmbH) in each quarter for 2 days. The heifers were moved to a separate tie-stall barn 48 h prior to inoculation, and kept there until the end of the experiment (i.e. 78 h after inoculation). The heifers were milked twice a day, at 08:00 and 20:00 h. After milking, the teats were dipped with an iodine-based barrier dip (Io-Shield, Ecolab, Northwich, UK) and before sampling, the teats were cleansed with a lactic acid based foam product (Oxy-Foam D, Ecolab, Northwich, UK).

### Study design

All heifers were challenged following a split-udder design [20, 21]. Three quarters of each heifer were simultaneously inoculated with 1.0 × 10^6^ colony forming units (CFU) of the bacterial strains (one strain per udder quarter) in 5 mL sterile phosphate-buffered saline (PBS) using a sterile polyvinyl chloride catheter of 18 cm. The remaining quarter was inoculated in the same manner with 5 mL sterile PBS (Thermo Scientific, Waltham, USA) and served as a control. All inocula were directly infused into the gland cistern. To ensure a balanced distribution between the quarter positions, the inocula were allocated to the quarters using restricted randomization. The heifers were examined clinically and their rectal temperature was registered at each sampling.

### Inocula

Three different CNS strains were used in this experimental challenge (Table 1). Two field strains of *S. chromogenes* were used; one isolated from the teat apex of a heifer with no signs of mastitis (*S. chromogenes* TA) [19] and the other from the left hind quarter of a multiparous cow with a persistent IMI lasting over 300 days (*S. chromogenes* IM) [9]. Although no genotypic strain typing was performed, there were notable phenotypic differences between both *S. chromogenes* isolates (Table 1). For instance, the bacterial inhibitory capacities of the present strains were tested against a field isolate of *S. aureus* in a previously described modified cross-streaking culture [19]. *Staphylococcus chromogenes* TA is able to inhibit the growth of *S. aureus*, whereas *S. chromogenes IM* is not. Both strains elicit a different immune response in mice [22] and interact differently with mammary epithelial cells [23]. The third CNS strain used in this study was *S. fleurettii* [9]. The strains were initially stored at −80 °C (Microbank, Pro-Lab Diagnostics, UK). A growth curve was set up for each strain by incubating one colony in brain–heart infusion broth at 37 °C. The bacteria were collected during the late logarithmic growth phase. The bacteria were washed three times in sterile PBS by centrifugation at 4000 × *g* for 10 min. The pellet was resuspended in PBS with 15% (v/v) glycerol and stored at −80 °C. To confirm the viable bacterial count in the stock solution, serial dilutions were plated on tryptic soy agar (TSA, Oxoid, Basingstoke, UK). An infection dose of 1.0 × 10^6^ CFU was selected based on the results of a preliminary challenge trial to induce subclinical mastitis rather than clinical mastitis [21].

**Table 1:** **The characteristics of the coagulase-negative staphylococcal isolates used in this study**

Strain	Origin	Colony characteristics				References
		Color	Shape	Consistency	In vitro growth inhibition of *S. aureus*	
*S. fleurettii*	Sawdust	Grey	Round	Creamy	No	[9]
*S. chromogenes* (teat apex; TA)	Heifer’s teat apex	Beige	Round	Creamy	Yes	[19]
*S. chromogenes* (intramammary; IM)	Intramammary infection in a cow lasting >300 days	Orange	Round	Creamy	No	[9]

### Milk samples

#### Collection

Milk samples were collected aseptically from the CNS-challenged and control quarters in duplicate 24 h before inoculation (bi) and at 0, 4, 6, 9, 12, 18, 24, 28, 32, 36, 48, 54, 60, 72 and 78 h post-inoculation (pi) for microbiology, SCC and cytokine measurements. The apoptosis and necrosis of polymorphonuclear neutrophil leukocytes (PMN) was determined 24 h bi and at 0, 6, 12, 18, 24, 48 and 72 h pi. Additional milk samples were collected after the actual experiment, at 144 and 216 h pi, to evaluate the progression of bacterial shedding and quarter milk SCC. Milk samples were kept on ice during transportation to the laboratory of Mastitis and Milk Quality Research Lab (Faculty of Veterinary Medicine, Ghent University, Merelbeke, Belgium).

#### Microbiology and somatic cell counts

The SCC was determined by a DeLaval Cell counter (DeLaval, Tumba, Sweden). The milk samples (10 µL) were plated in duplicate on aesculin-blood and MacConkey agar (Oxoid, Basingstoke, UK) according to the guidelines of the National Mastitis Council [24]. The plates were incubated at 37 °C in aerobic conditions, and examined after 24 and 48 h. Additionally, serial dilutions of the milk were plated in duplicate on TSA for counting the CFU. Every morphologically dissimilar colony type on TSA was collected, and subjected to Gram staining, together with catalase, DNAse and tube coagulase testing (i.e. the routine identification methods for CNS). One colony of each morphologically dissimilar Gram-positive, catalase-positive and coagulase-negative isolate was stored at −80 °C (Microbank, Pro-Lab Diagnostics, Richmond Hill, Canada) and subjected to transfer RNA-intergenic spacer PCR (tDNA-PCR) for further identification at the species level [5]. Only if the isolates could not be identified using tDNA-PCR, *rpoB* sequencing was carried out [25].

#### Apoptosis and necrosis of milk PMN

The apoptosis and necrosis of the milk PMN, considered an indirect indicator of their impaired functionality [26], were determined 24 h bi and at 0, 6, 12, 18, 24, 48 and 72 h pi by means of flow cytometry [27]. This dual staining technique with annexin V-fluorescein isothiocyanate (FITC) and propidium iodide (PI) differentiates the (early) apoptotic (FITC+/PI−) and necrotic (FITC+/PI+) PMN from the intact, viable cells (FITC−/PI−). The raw data were acquired and analyzed with FACSDiva Software (BD Biosciences, San Jose, USA).

#### Cytokine measurement

The cytokines interleukin 1 beta (IL-1β) and tumor necrosis factor alpha (TNF-α), together with the chemokine interleukin 8 (IL-8) were measured. First, the fresh milk samples were centrifuged at 16 000 × *g* for 30 min at 4 °C (Centrifuge 5418R, Eppendorf, Hamburg Germany). The fat-depleted whey fraction was stored at −80 °C. The concentration of IL-1β, IL-8 and TNF-α was determined by sandwich ELISA. A commercially available kit was used to measure IL-8 (DY208, R&D Systems, Minneapolis, USA) and IL-1β (ESS0027, Thermo Scientific, Waltham, USA) according to the manufacturer’s instructions. The TNF-α measurement was based on another study [28] (MCA2334, PBP005 and MCA2335B, AbD Serotech, Oxford, UK).

### Statistical analysis

The entire data of one quarter were not included in the final analysis due to an elevated SCC at the moment of inoculation. The data of another quarter were also omitted from the analysis due to a naturally occurring IMI with *Staphylococcus epidermidis*, but only 12 h after the inoculation.

Linear mixed regression analysis was used to model the relationship between the inoculum (categorical variable: control, *S. fleurettii*, *S. chromogenes* IM and TA), the time of sampling (continuous variable: from 4 h until 78 h pi), the quadratic term of time of sampling (continuous variable) and the different outcome variables (bacterial count, SCC, % apoptotic milk PMN, and % necrotic milk PMN) (PROC MIXED, SAS 9.4, SAS Institute Inc.). The bacterial count was log_10_-transformed, whereas the % apoptotic PMN, % necrotic PMN, and SCC/µL underwent a natural logarithmic transformation to obtain a normalized distribution. Heifer and quarter were incorporated as random effects in every model to account for the correlated nature of the data. Compound symmetry was selected as a covariance pattern to correct for the clustering of multiple samplings per quarter. To emphasize the response after challenge, measurements prior to inoculation were not included in the analysis. The interaction between inoculum and time of sampling was tested each time, and kept in the model when significant. The significance level was set at *P* ≤ 0.05. For pairwise comparisons between the different bacterial strains and control, a Bonferroni adjustment was used.

## Results

### Clinical parameters

None of the inoculated quarters showed physical signs of clinical mastitis during the trial. A short period of fever was observed in three heifers though (>39.5 and <40.6 °C) between 9 and 12 h pi.

### Somatic cell count

**Figure 1:**
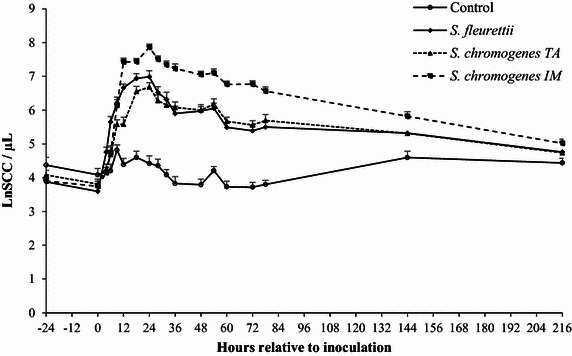
**Average somatic cell count (SCC).** The SCC is expressed as a natural logarithm (Ln SCC/µL) before and after intramammary challenge with *Staphylococcus fleurettii*, *S. chromogenes* TA (teat apex strain), *S. chromogenes* IM (intramammary strain) and phosphate-buffered saline (control). The error bars represent the standard error of the mean (+SEM).

The milk SCC was significantly higher in the quarters inoculated with any of the isolates compared to the control quarters (adjusted *P* < 0.01; Table 2). No significant difference was found between the SCC response in quarters challenged with *S. fleurettii*, and those challenged with either *S. chromogenes* strain (*S. chromogenes* IM and TA: adjusted *P* = 0.43 and *P* = 1.00). However, the SCC tended to be more pronounced in the quarters challenged with *S. chromogenes* IM than with *S. chromogenes* TA (adjusted *P* = 0.06). The evolution over time of the quarter milk SCC after challenge differed significantly between quarters (interaction inoculum x time of sampling: *P* < 0.01; Table 2). After 78 h, the SCC continued to decline in the challenged quarters (Figure 1).

**Table 2:** **Linear mixed regression model for somatic cell count, bacterial count, apoptotic and necrotic neutrophils**

Predictor variables	Ln SCC (cells/µL)				Log (CFU/mL + 1)				Ln apoptotic PMN (%)				Ln necrotic PMN (%)			
	β^a^	SE^b^	LSM^c^	*P*	β	SE	LSM	*P*	β	SE	LSM	*P*	β	SE	LSM	*P*
Intercept	3.43	0.34	–	<0.01^d^	0.52	0.16	–	<0.01^d^	3.16	0.19	–	<0.01^d^	2.72	0.11	–	<0.01^d^
Inoculum				<0.01^d^				<0.01^d^				<0.01^d^				<0.01^d^
Control	Ref.^e^	–	3.91	–	Ref.	–	−0.03	–	Ref.	–	3.48	–	Ref.	–	3.15	–
* S. fleurettii*			6.07	<0.01^f^			0.22	0.25^f^			2.85	0.01^f^			2.79	0.03^f^
* S. chromogenes* TA			5.78	<0.01^f^			0.50	<0.01^f^			3.06	0.13^f^			2.90	0.29^f^
* S. chromogenes* IM			6.71	<0.01^f^			0.89	<0.01^f^			2.92	0.02^f^			2.85	0.10^f^
Time of sampling	0.06	0.01	–	<0.01^d^	−0.05	0.01	–	<0.01^d^	0.01	0.00	–	<0.01^d^	0.02	0.15	–	<0.01^d^
Quadratic term of time of sampling	0.00	0.00	–	<0.01^d^	0.00	0.00	–	<0.01^d^				NS^g^				NS
Inoculum × time of sampling	–	–	–	<0.01^d^	–	–	–	<0.01^d^	–	–	–	0.02^d^	–	–	–	0.01^d^

^a^Regression coefficient.

^b^Standard error.

^c^Least square means.

^d^ *P* value for overall effect.

^e^Reference.

^f^Bonferroni-adjusted *P* value for comparing different inocula with control.

^g^Not significant.

### Bacterial shedding

**Figure 2:**
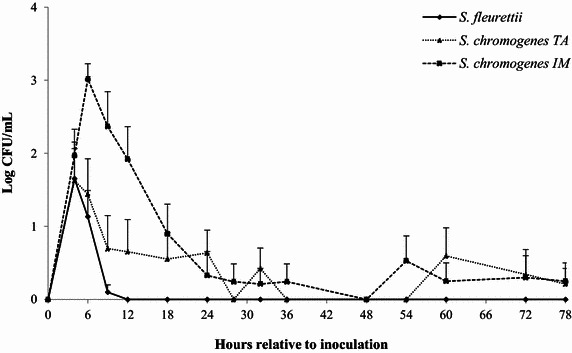
**The average bacterial count.** The bacterial count (log CFU/mL) was determined after experimental intramammary challenge with *Staphylococcus fleurettii*, *S. chromogenes* TA (teat apex strain), *S. chromogenes* IM (intramammary strain) and phosphate-buffered saline (control). The error bars represent the standard error of the mean (+SEM). The control quarters remained culturally negative throughout the study.

The control quarters remained culture-negative throughout the trial. The bacterial shedding was significantly higher in the *S. chromogenes* TA- and *S. chromogenes* IM-challenged quarters than in the control quarters (adjusted *P* values < 0.01). It was significantly more pronounced in *S. chromogenes* IM than in *S. chromogenes* TA (adjusted *P* ≤0.01). The bacterial shedding was so low in the *S. fleurettii*-inoculated quarters, that there was no significant difference with the control quarters (adjusted *P* value = 0.25). The evolution over time of bacterial shedding differed significantly between quarters (interaction inoculum x time of sampling: *P* < 0.01; Table 2). In each cow, *S. fleurettii* was eliminated from the mammary gland within 12 h, whereas *S. chromogenes* IM and TA remained present for a longer period of time (Figure 2). No CNS were found in the inoculated quarters after 144 h pi though.

### Milk PMN apoptosis and necrosis

**Figure 3:**
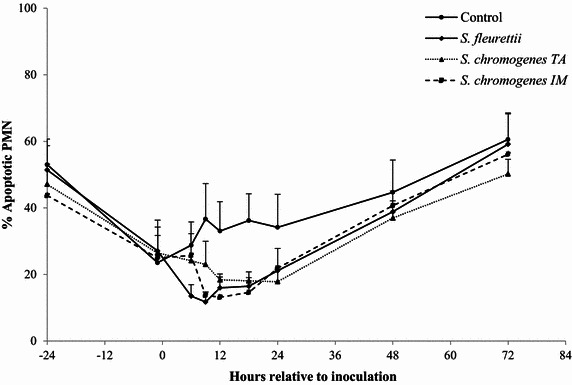
**Proportion of apoptotic neutrophils.** The proportion of apoptotic neutrophils (% apoptotic PMN) was determined in quarter milk before and after experimental intramammary challenge with *Staphylococcus fleurettii*, *S. chromogenes* TA (teat apex strain), *S. chromogenes* IM (intramammary strain) and phosphate-buffered saline (control). The error bars represent the standard error of the mean (+SEM).

**Figure 4:**
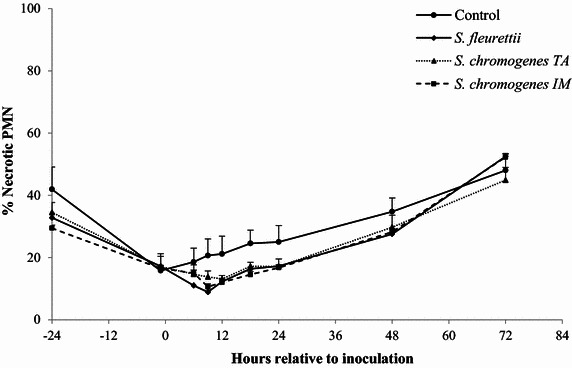
**Proportion of necrotic neutrophils.** The proportion of necrotic neutrophils (% necrotic PMN) was determined in quarter milk before and after experimental intramammary challenge of eight dairy heifers with *Staphylococcus fleurettii*, *S. chromogenes* TA (teat apex strain), *S. chromogenes* IM (intramammary strain) and phosphate-buffered saline (control). The error bars represent the standard error of the mean (+SEM).

The highest proportion of apoptotic and necrotic PMN was found in the control quarters. Compared to the control quarters, only the *S. fleurettii*- and *S. chromogenes* IM-challenged quarters yielded significantly less apoptotic milk PMN (adjusted *P* value <0.01 and 0.02 respectively; Table 2). No significant difference was found in the number of apoptotic PMN between the control quarters and *S. chromogenes* TA (adjusted *P* value = 0.13). *Staphylococcus fleurettii* was the only strain that resulted in significantly less necrotic PMN than in the control quarters (pairwise comparison: *P* = 0.03; Figure 3). No significant differences were found when comparing the different CNS isolates with each other in terms of PMN apoptosis or necrosis. The evolution over time of the proportion of apoptotic and necrotic PMN differed significantly between quarters though (interaction inoculum x time of sampling: *P* = 0.02 and *P* = 0.01; Table 2). It should also be noted that the proportion of apoptotic and necrotic PMN was reduced in all quarters immediately before the inoculation, compared to 24 h in advance (Figures 3 and 4).

### Milk IL-1β, IL-8 and TNF-α

**Figure 5:**
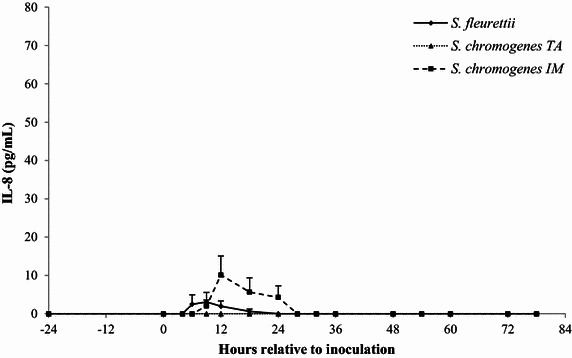
**The average concentration of IL-8.** IL-8 (pg/mL) was determined in quarter milk before and after experimental intramammary challenge with *Staphylococcus fleurettii*, *S. chromogenes* TA (teat apex strain), *S. chromogenes* IM (intramammary strain) and phosphate-buffered saline (control). The error bars represent the standard error of the mean (+SEM). IL-8 was not detected in the control quarters.

**Figure 6:**
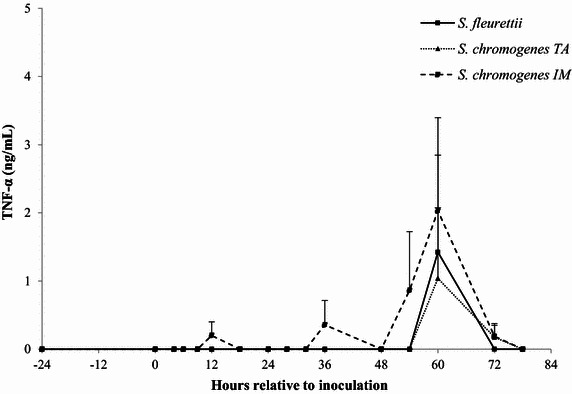
**The average concentration of TNF-α.** TNF-α (ng/mL) was determined in quarter milk before and after experimental intramammary challenge with *Staphylococcus fleurettii*, *S. chromogenes* TA (teat apex strain), *S. chromogenes* IM (intramammary strain) and phosphate-buffered saline (control). The error bars represent the standard error of the mean (+SEM). TNF-α was not detected in the control quarters.

**Figure 7:**
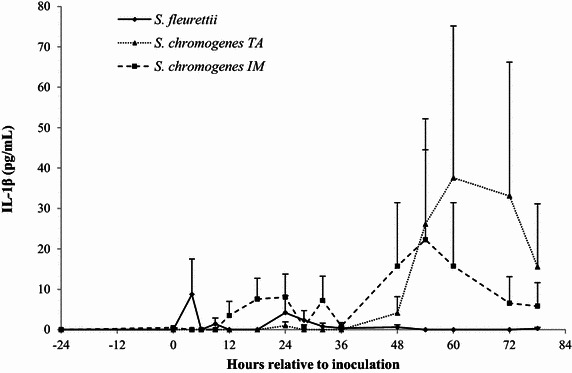
**The average concentration of IL-1β.** IL-1 β (pg/mL) was determined in quarter milk before and after experimental intramammary challenge with *Staphylococcus fleurettii*, *S. chromogenes* TA (teat apex strain), *S. chromogenes* IM (intramammary strain) and phosphate-buffered saline (control). The error bars represent the standard error of the mean (+SEM). IL-1β was not detected in the control quarters.

The IL-8 and IL-1β ELISA had an intra- and inter-assay coefficient of variation (CV) of <6 and <15%, respectively. The TNF-α ELISA showed lower precision (intra- and inter CV: 13 and 28%, respectively). Since the inoculation of the CNS strains generally elicited a minimal, or non-detectable cytokine response, the IL-1β, IL-8 and TNF-α levels were not statistically analyzed. Instead, descriptive statistics were generated. In the *S. fleurettii*- and *S. chromogenes* IM-challenged quarters, a low, transient IL-8 response (<30 pg/mL) was observed within 28 h after inoculation (Figure 5). These quarters also showed limited, erratic TNF-α peaks (<10 ng/mL; Figure 6). *Staphylococcus chromogenes* IM caused a higher, long-lasting IL-1β response compared to the TNF-α response, starting at 12 h pi (Figure 7). *Staphylococcus chromogenes* TA did not evoke a detectable IL-8 response, but the strain caused a major increase in IL-1β (peaking at 300 pg/mL 60 h pi) in one heifer. This particular heifer was also the only animal that demonstrated a TNF-α reaction to *S. chromogenes* TA peaking at 60 h pi. Notably, the data of the *S. fleurettii* quarter of the same heifer were omitted from the analysis 12 h pi due to the occurrence of a spontaneous, natural infection in that quarter. Another heifer showed no discernable cytokine response in any challenged quarter. No cytokines were found in the control quarters.

## Discussion

First, we wanted to compare the host responses in dairy cattle with subclinical mastitis caused by representative isolates of either an “environmental” or a “host-associated” CNS species. For this purpose, *S. fleurettii* was selected as the environmental species, and *S. chromogenes* as the host-adapted species. The second objective was to study the host’s reaction to different strains of the same species, in this case *S. chromogenes*. Due to the split-udder design, which partially circumvents variation between heifers, the host response could be studied using only a limited number of experimental animals. In contrast to other experimental CNS trials [28, 29], all CNS species found in the milk samples were identified with tDNA-PCR, in addition to conventional culturing techniques. Through the molecular identification of the CNS species, a natural infection with *S. epidermidis* was detected. The data of this particular quarter were subsequently discarded from the analysis.

Based on the changes in SCC and bacterial shedding, all quarters were successfully challenged with the different CNS strains, whereas the control quarters remained culture-negative throughout the entire trial. *Staphylococcus fleurettii* was fairly quickly (i.e. within 12 h) eliminated from the mammary gland, and the bacterial shedding was lowest for this species. The latter finding was also observed in an experimental trial in mice using the same CNS isolates [22]. Both *S. chromogenes* strains, on the other hand, persisted in the mammary gland for at least 3 days, accentuating the host-adapted nature of this species. Altogether, this is still a short period, considering that *S. chromogenes* IM was originally isolated from a cow suffering from a persistent IMI lasting over 300 days [9]. The bacterial count of *S. chromogenes* TA was significantly lower than *S. chromogenes* IM though. In fact, *S. chromogenes* IM seemed to be the only strain able to multiply in the mammary gland in the first 6 h after inoculation. The *S. chromogenes* IM strain tended to elicit a larger increase in SCC than *S. chromogenes* TA. The lower cellular response in the *S. chromogenes* TA-infected quarters could possibly explain the slower bacterial clearance. These results might indicate a difference in pathogenicity and in vivo growth capacity between the different strains. Interestingly, in the murine experimental trial, no differences in bacteriological shedding nor in neutrophil influx were observed between *S. chromogenes* IM and *S. chromogenes* TA [22].

The intramammary challenge had a significant effect on the apoptosis of PMN in milk, which was likely the result of an influx of young, activated PMN from the blood stream to the site of infection along with a potentially delayed PMN apoptosis. Aging PMN eventually undergo apoptosis (or programmed cell death), impairing the functionality of the cells [30, 31]. Delayed apoptosis might therefore contribute to a faster clearing of the bacterial infection [26]. However, PMN apoptosis and subsequent phagocytosis by macrophages is a physiological necessity to curb unbridled inflammatory responses that result in tissue damage [30]. Since the lactation stage and parity may affect the survival of PMN [26, 32], only mid-lactation heifers were used in this experiment, allowing us to study the effect of the infection stage [33]. *Staphylococcus fleurettii* evoked the greatest decrease in PMN apoptosis during the first 48 pi, at least partly explaining the higher (albeit insignificant) SCC increase compared to *S. chromogenes* TA. Still, the PMN apoptosis was already decreased in all quarters right before the inoculation. The pre-inoculation drop in apoptosis might have been caused by stress [34] associated with the beginning of the experiment, although this could not be confirmed. A longer adaptation period (>48 h) in the tie-stall facility prior to the inoculation might have mitigated these findings.

Pro-inflammatory cytokines, such as TNF-α and IL-1β, are involved in a plethora of immune functions on a local and systemic level (e.g. the endothelial adhesion of immune cells, induction of fever or the production of other cytokines) [35, 36]. The further recruitment of PMN to the infection site is mediated by IL-8 and other chemokines [37]. In contrast to *Escherichia coli* infections, *S. aureus* mastitis does not evoke a significant IL-8 or TNF-α response, which might partially explain the chronic nature of *S. aureus* IMI [38, 39]. As demonstrated in previous research [28], other CNS species (*S. epidermidis* and *Staphylococcussimulans*) appear to induce a clear pro-inflammatory response with TNF-α, IL-8 and IL-1β nonetheless.

In this study, *S. chromogenes* IM induced the largest overall pro-inflammatory cytokine response, starting with an increase in IL-8 at 9 h pi. The production of IL-1β occurred later at 12 h pi, but lasted longer (>78 h pi). The IL-1β response was less pronounced in the *S. fleurettii*-challenged quarters than in the *S. chromogenes* IM-quarters. The IL-8 response induced by these strains seemed to be smaller than the response described for *S. epidermidis* or *S. simulans* [28]. However, a higher infection dose was used in the latter study. Furthermore, the authors transformed the IL-8 data by multiplying it by 100, since they observed that the human IL-8 ELISA measures bovine IL-8 100-times less efficiently. That might explain why their IL-8 results fall in the ng/mL range, whereas our results are found in the pg/mL range. Also, we did not find a distinction between early (peaking at 12 h) or late (peaking at 30 h) IL-8 responders as seen in [28]. In fact, none of our animals displayed any IL-8 response after 30 h pi.

*Staphylococcus chromogenes* TA did not elicit any detectable cytokine response, except in one particular heifer. In a similar murine infection trial, some mice displayed a disproportionate reaction to *S. chromogenes* TA as well, resulting in a high IL-1β response and an intense clinical reaction [22]. Another heifer showed no cytokine response at all to any CNS strain. Everything considered, the pro-inflammatory cytokine response appeared to vary greatly between individual animals in our research.

As discussed in other research [40], the split udder design used in this experiment is founded on within-cow comparisons. On the one hand, this study design partly circumvents high variation between individual animals, and reduces the needed number of research animals substantially. On the other hand, it assumes that all mammary quarters are completely separate anatomical entities within the udder. This is not necessarily the case, as demonstrated by previous research that illustrated that the immune response of the neighboring (uninfected) quarters is affected by an experimental challenge with major mastitis pathogens [41]. This should be kept in mind when interpreting the results of this study. Nonetheless, we were still able to demonstrate different host responses to each CNS strain while using the split-udder design.

None of the heifers in our study showed any local signs of clinical mastitis (such as clots in the milk or swelling of the quarters), although 3 animals experienced a brief bout of fever in the first 12 h after inoculation. In a similar challenge study with *S. chromogenes*, the heifers only developed very mild clinical signs of inflammation, even though the inoculation dose was more than double ours (2.1 × 10^6^ versus 1.0 × 10^6^ CFU) [29]. In the preliminary challenge trial conducted prior to this study [21], higher doses of the same *S. chromogenes* strain evoked a more pronounced immune response as opposed to lower doses with the appearance of mild clinical signs at a dose of 2 × 10^6^ CFU. Observational research has demonstrated that approximately half of all cows with an intramammary *S. chromogenes* infection showed clinical signs of mastitis [11, 42]. Notwithstanding a different infection dose or host-immune status, this could suggest that not all *S. chromogenes* strains are equally pathogenic. This is in accordance with *S. aureus*, where certain strains have also been linked to a more severe clinical outcome, with a reduced persistence in the mammary gland [43]. When extrapolating the current results to practice, it should be noted that natural infection doses might vary from doses used here, and that the experimental strains were directly infused into the teat cistern (as opposed to natural infections, where CNS have to overcome the teat barrier.) The results of our experimental trial with dairy heifers should be interpreted with caution, since the outcome of an IMI may vary with parity, lactation stage and other cow factors.

Even in case of large inoculation doses, bovine-associated *S. chromogenes* and environmental *S. fleurettii* strains trigger a similar, relatively mild local response. The environmental CNS species, *S. fleurettii*, evokes a profound cellular response in dairy heifers nonetheless, akin to the host-adapted species *S. chromogenes*. However, *S. fleurettii* is eliminated more rapidly from the mammary gland than *S. chromogenes*. This might indicate that certain bovine-associated CNS species, like *S. chromogenes*, are better able to withstand and thrive in the mammary gland. The present in vivo study also suggests that not all *S. chromogenes* strains exhibit the same degree of pathogenicity. The clinical outcome of a natural *S. chromogenes* mastitis might therefore not only depend on the infection pressure and the resistance of the cow, but also on the pathogenicity of the particular strain.
